# A Core Regulatory Circuit in Glioblastoma Stem Cells Links MAPK Activation to a Transcriptional Program of Neural Stem Cell Identity

**DOI:** 10.1038/srep43605

**Published:** 2017-03-03

**Authors:** Gregory Riddick, Svetlana Kotliarova, Virginia Rodriguez, H. S. Kim, Amanda Linkous, Andrew J. Storaska, Susie Ahn, Jennifer Walling, Galina Belova, Howard A. Fine

**Affiliations:** 1 National Cancer Institute, Neuro-Oncology Branch, Bethesda, MD, USA; 2 Macrogen, Seoul, Korea; 3 Weill Cornell Medical College, New York, NY, USA

## Abstract

Glioblastoma, the most common primary malignant brain tumor, harbors a small population of tumor initiating cells (glioblastoma stem cells) that have many properties similar to neural stem cells. To investigate common regulatory networks in both neural and glioblastoma stem cells, we subjected both cell types to *in-vitro* differentiation conditions and measured global gene-expression changes using gene expression microarrays. Analysis of enriched transcription factor DNA-binding sites in the promoters of differentially expressed genes was used to reconstruct regulatory networks involved in differentiation. Computational predictions, which were biochemically validated, show an extensive overlap of regulatory circuitry between cell types including a network centered on the transcription factor KLF4. We further demonstrate that EGR1, a transcription factor previously shown to be downstream of the MAPK pathway, regulates KLF4 expression and that KLF4 in turn transcriptionally activates NOTCH as well as SOX2. These results demonstrate how known genomic alterations in glioma that induce constitutive activation of MAPK are transcriptionally linked to master regulators essential for neural stem cell identify.

Glioblastoma is one of the most aggressive and frequent primary brain tumors, with a median survival time of 14–16 months. Several lines of evidence support neural stem cells as the target cell where initiating genomic alterations first emerge. Neural stem cells (NSC) are primarily located in the Subventricular Zone (SVZ) region of the brain, which is a common site of origin for glioma^1^. Pathways known to have roles in NSC development, including activation of AKT, RAS/ERK, BMI-1, NOTCH, and WNT, frequently show genomic alteration or aberrant activation in glioblastoma. Glioblastoma stem-cells (GSC) isolated from tumor tissue retain the ability to form tumors, but show characteristics of NSCs, including self-renewal and multipotency^2^,^3^,^4^,^5^. In addition, GSCs express many of the characteristic markers of NSCs including SOX2, Nestin, CD133, and also demonstrate upregulation of GFAP during differentiation to an astrocytic lineage^6^.

Murine models of gliomagenesis have also supported the hypothesis that gliomas originate from normal neural stem cells. Mice with germline mutations and/or functional activation of Ras and AKT^7^, inactivation of NF1 and P53^8^, or inactivation of P53 and PTEN^9^ develop tumors from neural progenitor cells. This population of neural progenitor cells in the SVZ was subsequently shown to be the source of tumor regrowth in a mouse model after treatment with Temozolomide^10^. Work using mosaic analysis with double markers (MADM) further suggested that oligdendrocytic precursor cells derived from neural stem cells may be the sub-lineage in which the greatest clonal expansion occurs^11^.

GSC cultured in Neuralbasal Medium(NBE) media share the capacity of neural stem cells to differentiate into neural and/or glial lineages when media is changed to serum containing media(FBS) and retinoic acid(RA)^12^. A number of different studies have used global gene expression changes and other genome-wide assays during NSC differentiation to identify master regulators and pathways^13^,^14^. In the most comprehensive analysis of NSC cis-regulatory elements to date, Mateo *et al*.^15^ combined DNASE-seq with an analysis of histone modifications to study cis regulatory elements(CRE) in neural stem cell biology to reveal a core network of transcription factors motifs including bHLH, NFI, SOX, and FOX. An in-depth analysis of the bHLH transcription factor Olig2 using ChIP-Seq confirmed the genomic analysis and computational predictions.

In a similar way, two recent efforts used global gene expression changes during GSC differentiation to identify 5 functionally important transcription factors: NOTCH1^16^, SOX2, SALL1, POU3F, and Olig2^17^ that act to block differentiation in GSC. Carro *et al*.^18^ also used global gene expression data from clinical glioma samples to infer the activity of master regulators STAT3 and CEBPB in the Mesenchymal subtype of glioblastoma and showed that these transcription factors could reprogram a glioma cell line towards a mesenchymal lineage^18^.

Given the importance of the proneural clinical subtype of glioblastoma, we asked whether differentiation-induced global gene expression changes in 5 GSC derived from 5 parental proneural tumors could reveal type-specific master regulators. Additionally, we sought to compare regulatory networks between proneural GSC and neural stem cells with the aim to elucidate how pathways affected by genomic alterations specific to glioma could act to induce constitutive activation of master regulators important for neural stem identity and self-renewal. Identification of a network of neural stem-related regulators in GSCs and how these regulators are connected to oncogenic pathways in glioma could potentially lead to new treatment strategies designed to induce differentiation in GSCs and halt tumor progression.

## Results

### Inferring Regulatory Networks During *In-Vitro* Differentiation

**Figure 1:**
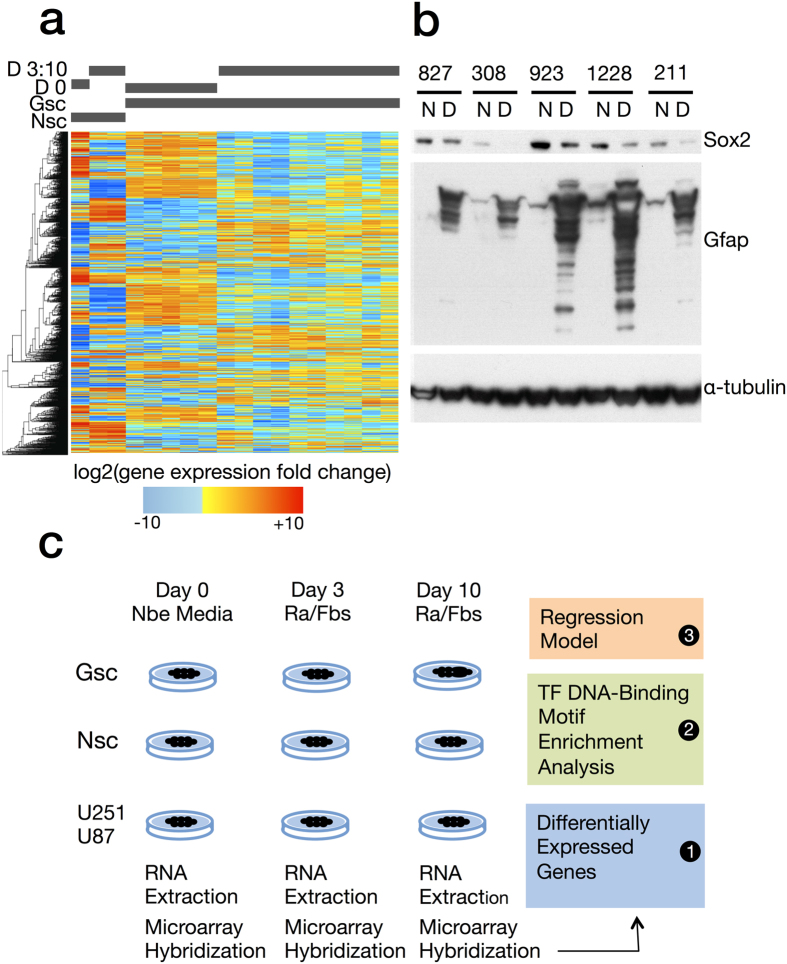
Global Gene Expression Changes During Differentiation in Neural and Glioblastoma stem cells. (**a**) 5 Glioblastoma stem cell lines and one murine neural stem cell (E14) were subjected to *in-vitro* differentiating conditions (Rentioic Acid/FBS). Total RNA was isolated from cell lysate at days 0, 3, and 10 of the differentiation experiment. Global gene expression was assayed using Affymetrix 133 plus 2.0 microarray. Significant differentially expressed genes across days 0, 3, and 10 (2-way ANOVA, fdr < 0.05) are shown in a hierarchically clustered heatmap. (**b**) Markers of differentiation, SOX2 and GFAP, are shown by western blot for days 0 and 10 of the differentiation experiment (Images are cropped from the original, see Supplementary Data). (**c**) Schematic overview of the experimental and computational pipeline.

We developed a strategy to reconstruct regulatory networks in GSC and NSC from *in-vitro* differentiation experiments. Affymetrix microarrays were used to measure global gene expression changes in five different GSCs lines and one embryonic murine neural stem cell line (E14) in NBE media (day0) and after a switch to FBS/RA media (day3 and day10). Markers of differentiation (GFAP) and the undifferentiated state (SOX2 and Nestin) showed changes across 10 days of the experiment (Fig. 1).

**Figure 2:**
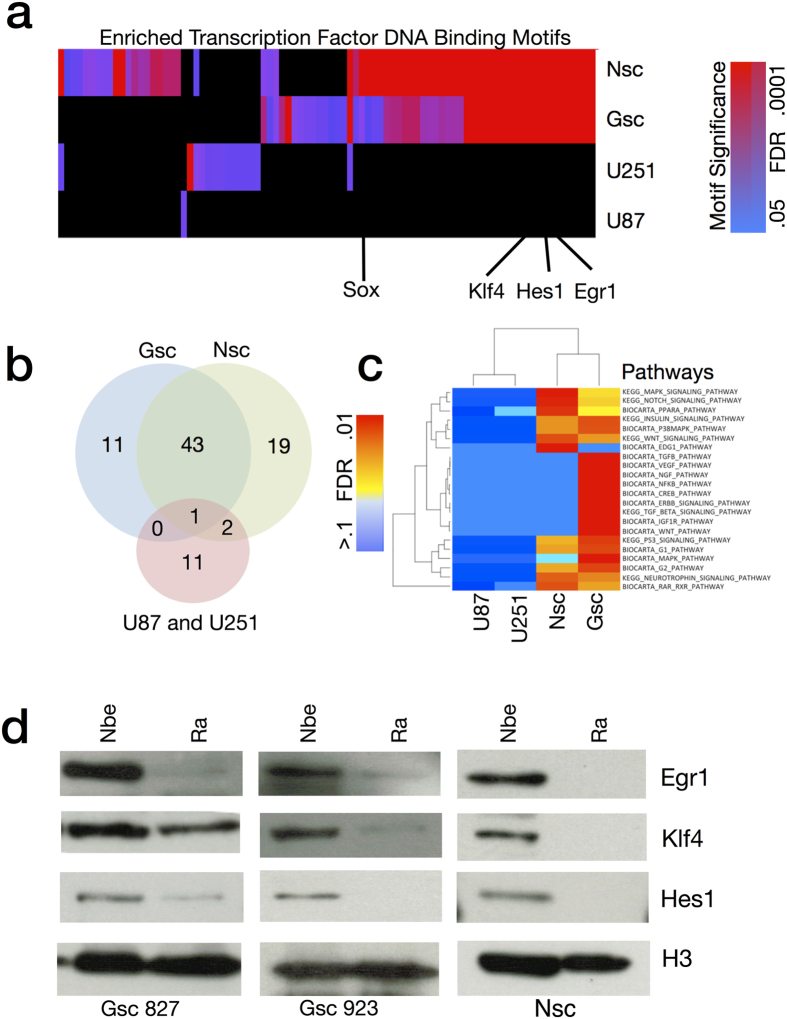
Functional Analysis of Global Gene Expression Changes during GSC Differentiation. (**a**) Activity of transcription factors was inferred by a motif enrichment analysis of the promoter regions of differentially expressed genes (2-way ANOVA fdr < 0.05) for 5 Glioblastoma Stem Cells, 1 murine neural stem cell (E14) and 2 established glioma cell-lines U251 and U87. (**b**) Overlap of significantly enriched motifs between GSC, E14, and the two established glioma cell-lines. (**c**) Pathway enrichment analysis of differentially expressed genes in 2 established glioma cell-lines, 1 murine neural stem cell line, and 5 glioblastoma cell-lines. (**d**) Validation of the transcription factors EGR1, KLF4, and HES1 by western blot of nuclear lysate for GSC827/GSC923 for both NBE media and after 3 days of differentiation by RA/FBS(Images are cropped from the original, see Supplementary Data).

**Figure 3:**
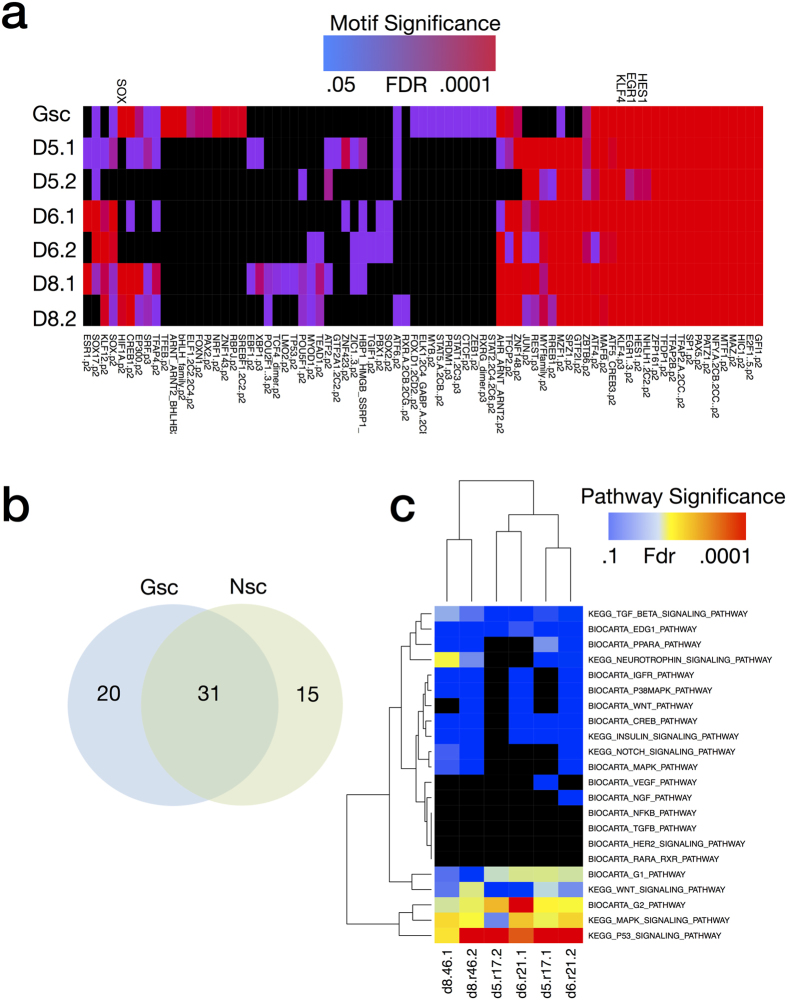
(**a**) Functional Analysis of Global Gene Expression Changes in Differentiation of 3 Primary Human Neural Stem Cell Lines. Transcription factor motif enrichment analysis of the promoters of differentially expressed genes of 3 primary human neural stem cells derived from brain tissue of 3 different donors (2 biological replicates each). (**b**) Overlap of significant transcription factor motifs between 5 GSC and 3 NSC. (**c**) Pathway enrichment analysis of 3 primary human neural stem cells.

To predict which transcription factors were active during differentiation, we next examined promoter regions of differentially expressed genes for the presence of known DNA-binding motifs of transcription factors (Fig. 2). Significant enrichment of these motifs over what would be expected by chance was used to infer activity of the cognate transcription factors. Results show a substantial overlap of predicted transcription factor activity between the 5 GSC lines and the NSC E14 during differentiation. Common transcription factors include those known to be important in NSC biology such as SOX2, KLF4, EGR1, HES1 (activated by NOTCH), and OLIG1/2. Further analysis of 3 neural stem cell lines derived from primary human brain tissue agreed with the findings from NSC E14 in the identification of a core set of transcription factor binding motifs representing SOX, EGR1, HES1, and KLF4 (Fig. 3)^19^. The transcription factor motif analysis results also overlapped with the results of a recent computational analysis of NSC cis regulatory elements (Mateo *et al*.^15^) including SOX, FOX, E2F, ETS, and HLH families as well as CTCF, MAX, Tfap2a, TCF, Sp1, Rest, and Zic.

Transcription factors specific to the GSCs include known oncogenes such as STAT3 (downstream of PI3K) and SRF (downstream of RAS) as well as suspected oncogenes such as FOXD1. Transcription factors specific to the NSCs included known tumor suppressors such as P53 and suspected tumor suppressors such YY1. Several key regulators, including SOX2, KLF4, EGR1, and HES1 were shown by western blot to exhibit protein expression in the nucleus that decreases with differentiation (Figs 1b and 2d). Established glioma cell lines U87 and U251, which are cultured in serum-containing media, showed minimal gene expression changes under the differentiation stimulus consistent with their non-stem cell nature (Fig. 2b).

To gain insight into the regulation of signaling pathways during GSC differentiation, we performed a geneset enrichment analysis of canonical pathways curated from the Biocarta and KEGG databases (Fig. 2c). Both NSC and GSC show changes in pathways related to the differentiation stimulus (RA/FBS), including retinoic acid signaling, downregulation of G1/G2 pathways, consistent with cell-cycle arrest, and a number of genesets associated with receptor tyrosine kinase signaling, such as EGFR, PDGFR, MAPK, ERK, AKT, and MTOR. Importantly, both cell types show downregulation of pathways associated with NSCs, including NOTCH, WNT, and Neurotrophin signaling. In addition, GSCs show changes in regulation to pathways previously associated with glioma, including VEGF, NF-kB, and TGF-Beta.

To connect the inferred activity of transcription factors to changes in signaling pathways and other regulators, a regression model of significantly enriched motifs vs. differentially expressed genes was created. Briefly, a N × M matrix of transcription factor binding motif scores was used to fit a multiple regression model against the set of N gene expression values that were found previously to be differentially expressed during *in-vitro* differentiation. Significant motif-target gene interactions defined by a regression model were identified as significant by the use of an empirical null distribution created by random permutation of both the motif scores and targets genes in the model (see methods).

### A Core Transcriptional Circuit Links EGR1 to KLF4, NOTCH, and SOX2

**Figure 4:**
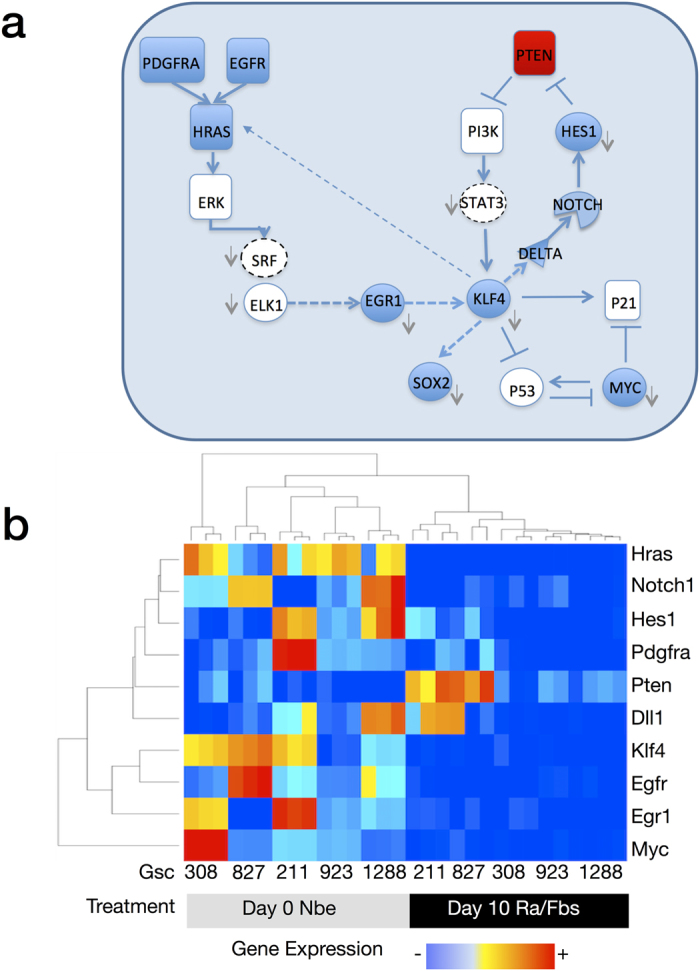
A Transcriptional Circuit Links MAPK activation to EGR1, KLF4, SOX2, and NOTCH. (**a**) A regression model was fit between significantly enriched transcription factor binding motifs and differentially expressed genes from the 5 GSCs in the differentiation experiment. Dashed lines represent regulatory relationships derived from the regression model. Solid lines represent relationships taken from the literature. The color of each regulator indicates direction of gene expression or protein level change during differentiation (blue for decreased expression and red for increased expression). Downward arrows indicate regulators that have significantly enriched motifs from the set of differentially expressed genes during differentiation. (**b**) Heatmap of gene expression changes in key regulators.

**Figure 5:**
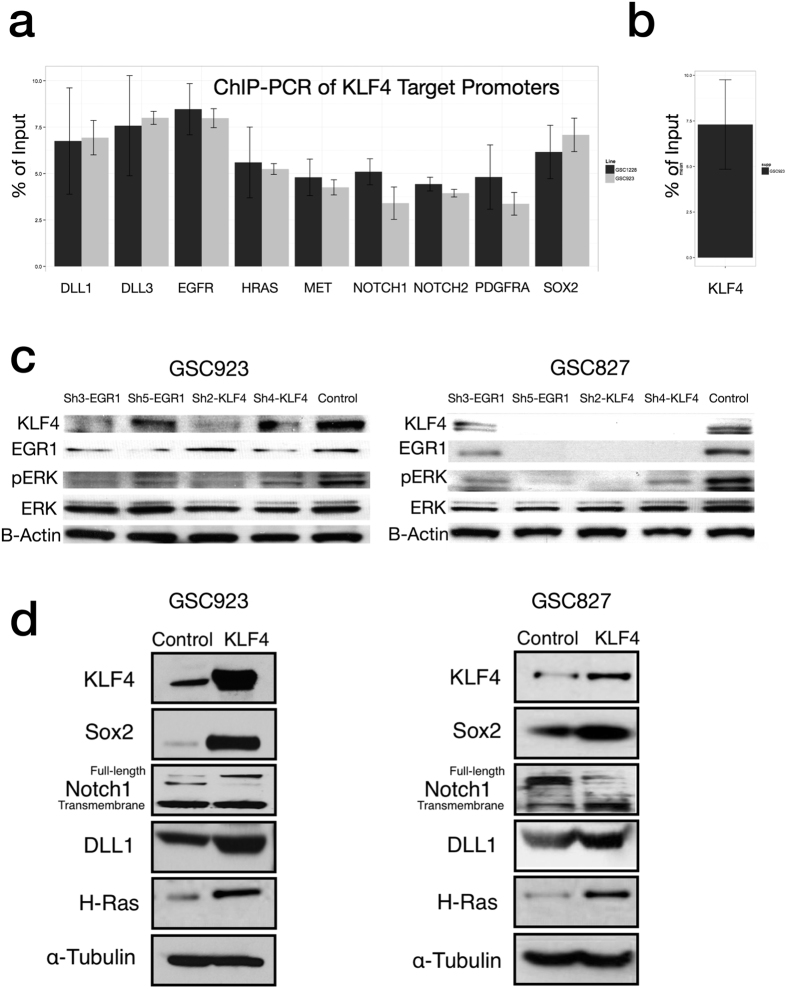
Transcriptional Regulatory Targets of KLF4 and EGR1. (**a**) ChIP-PCR of KLF4 against promoters of target genes. (**b**) ChIP-PCR of EGR1 against KLF4 promoter. (**c**) Knockdown of EGR1 reduces KLF4 and pERK levels in 2 glioblastoma stem cell-lines. (**d**) Western blot shows overexpression of KLF4 increases protein levels of SOX2, NOTCH1, DLL1, and HRAS(Images are cropped from the original).

Given the importance of the transcription factor KLF4 in NSC biology, we examined the resulting network of TF-Target-Gene interactions for both upstream and downstream regulators of KLF4 (Fig. 4). Upstream transcriptional activators of KLF4 predicted by the model included both STAT3 (a transcription factor previously shown to control KLF4 expression in embryonic stem cells) and EGR1 (a transcription factor and so-called intermediate early gene activated downstream of RAS/MAPK). The large decrease in EGR1 levels during GSC differentiation shown by both microarray (Fig. 4b) and by western blot (Fig. 2d) and the significant positive correlation between KLF4 and EGR1 gene expression levels in clinical GBM samples led us to examine the relationship between EGR1 and KLF4 protein levels. We constructed GSC827 and GSC923 cell lines stably expressing two different shRNAs directed against EGR1 and demonstrated significantly reduced levels of KLF4 (Fig. 5c). Binding of EGR1 to the upstream promoter region of KLF4 (−1 kb to 0 kb relative the TSS) was confirmed by ChIP-PCR of RNA purified from nuclear lysate in GSC827 incubated with an anti-EGR1 antibody (Fig. 5b). In addition, overexpression of EGR1 in U87 cells augmented the expression of KLF4 and vice versa (Supplementary Figure 2), No changes in neural stem cell markers were observed in U87, consistent with its non-stem cell nature.

RAS/MAPK activation in glioma has been shown to result both from constitutive activation of receptor tyrosine kinases EGFR, PDGFRA, and c-MET as well by an inactivating mutation of NF1, a suppressor of RAS GTPase activity. Given the well-established relationship between MAPK activation and EGR1 expression, we hypothesized that the transcriptional regulation of KLF4 by EGR1 in GSCs could act as a conduit through which known genomic alterations in glioma could be linked to the activation of master regulators of NSC identity and self-renewal. To test this hypothesis, we next examined the regulatory relationship in GSCs between KLF4 and both SOX2 and NOTCH1, two transcription factors important in NSC biology and previously shown to be under regulatory control of KLF4 in embryonic stem cells.

ChIP-PCR of DNA extracted from nuclear lysate by an antibody against KLF4 was used to identify promoters of genes bound by KLF4 in GSCs (Fig. 5a). Transcriptional activation of SOX2, DLL1(Notch ligand), and NOTCH1 predicted by the regression model was consistent with ChIP-PCR results *in-vitro*. Overexpression of KLF4 in GSCs also increased SOX2, DLL1, and NOTCH1 protein levels *in-vitro* as demonstrated by western blot (Fig. 5d).

### KLF4 forms a positive regulatory feedback loop with MAPK Activation

Having demonstrated the regulation of KLF4 by MAPK, we were next interested in exploring whether KLF4 could likewise regulate MAPK. First, we demonstrated that knockdown of KLF4 by shRNA reduced the phosphorylation of ERK (MAPK) by western blot (Fig. 5c). Since upstream regulators of RAS/MAPK activation including EGFR, PDGFRA, and HRAS were down-regulated following differentiation (Fig. 4b), we explored the regulatory relationship between KLF4 and the expression of these potential target genes. Binding of KLF4 to the upstream promoters (−1 kb, 0 kb TSS) of EGFR, HRAS, PDGFRA, and MET was confirmed by ChIP-PCR in GSC923 (Fig. 5a). However, overexpression of KLF4 induced only the upregulation of HRAS by western blot (Fig. 5d).

### Genome-Wide Binding of KLF4 in Glioblastoma Stem Cells

**Figure 6:**
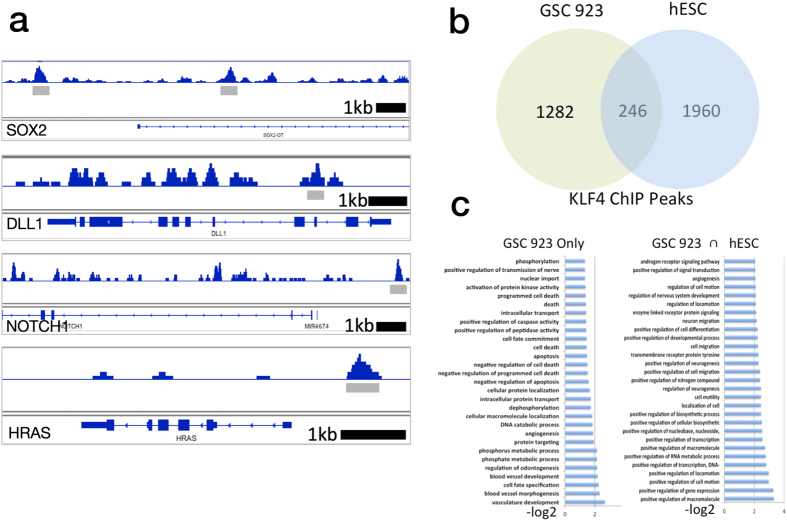
Genome Wide Binding of KLF4 in a Glioblastoma stem cell line measured by ChIP-Seq. (**a**) Binding of KLF4 near the promoters of SOX2, DLL1, NOTCH1, and HRAS. (**b**) Overlap of genes with promoters bound by KLF4 in Glioblastoma and hESC cell-lines. (**c**) Functional analysis of peaks bound by KLF4 in Glioblastoma and hESC cell lines.

KLF4 is known to play a major role in determining pluripotency in embryonic stem cells and was identified as one of the four original Yamanaka transcription factors. However, transcription factor binding can vary based on cell lineage and disease state. To study KLF4 binding in GSC, we performed a ChiP-Seq experiment to determine genome-wide binding. Out of 1528 genes with promoter binding sites identified, 246 overlap with KLF4 sites in embryonic stem cells (hESC) (Fig. 6b). A functional analysis of these peaks was performed using the annotation software GREAT^20^ (Fig. 6c). Peaks common to both GSC and hESC showed enrichment for pathways involved in neurogenesis including Regulation of Nervous System Development, Neuron Migration, and Positive Regulation of Neurogenesis. Pathways enriched specifically in GSC included terms relevant to glioma including Activation of Kinase Activity, Negative Regulation of Apoptosis, and several pathways involved in the positive regulation of angiogenesis. Figure 6a shows KLF4 binding peaks near the promoter regions of the genes SOX2, NOTCH1, DLL1, and HRAS.

### Increased KLF4 expression blocks GSC differentiation *in-vitro* and is associated with increased tumor aggressiveness *in-vivo*

**Figure 7:**
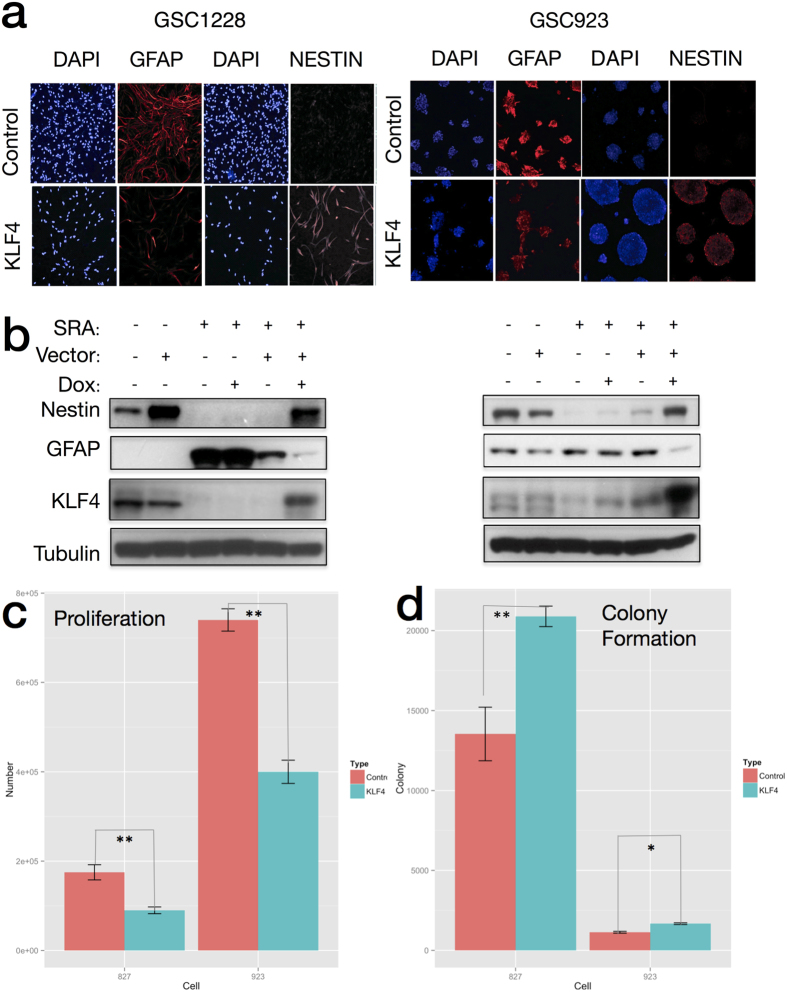
KLF4 overexpression blocks GSC differentiation by RA/FBS *in-vitro*. (**a**) Stable GSC cell-lines carrying a viral-transduced KLF4 expression vector or control (empty vector), were subjected to *in-vitro* differentiation using RA/FBS. Cells were immunostained for GFAP (a marker of differentiation) and Nestin (a marker of the undifferentiated state) in both NBE and RA/FBS conditions after 10 days. KLF4 overexpressing GSC lines show both lower GFAP and higher Nestin staining intensity after 10-day incubation with RA/FBS media compared to the control. (**b**) Stably transfected GSCs carrying a doxycycline-inducible vector retained nestin expression and lost GFAP expression intensity in the presence of doxycycline (KLF4 induction), as observed via western blot. The SRA condition indicates differentiating conditions by RA/FBS (Images are cropped from the original, see). (**c**) Overexpression of KLF4 decreases proliferation of glioblastoma stem cells relative to a control (p < 0.05, Students Independent T-Test, n = 6). (**d**) Overexpression of KLF4 increases clonogenic potential of glioblastoma stem cells in soft agar relative to a control (p < 0.05 Students Independent T-Test, n = 6).

To test the effect of KLF4 on GSC differentiation, we next stably overexpressed KLF4 in GSC lines 923 and 1228 and subjected GSCs to the differentiation stimulus (RA/FBS) for 12 days (Fig. 7a). Compared to the control (empty vector), KLF4 overexpression induced a blockade of differentiation, as seen by lower intensity staining of GFAP (marker of differentiation) and higher intensity staining of nestin (marker of the undifferentiated state) after incubation with RA/FBS for 10 days. These results were confirmed by western blot using an inducible Flag-tagged KLF4 expression system (Fig. 7b).

**Figure 8:**
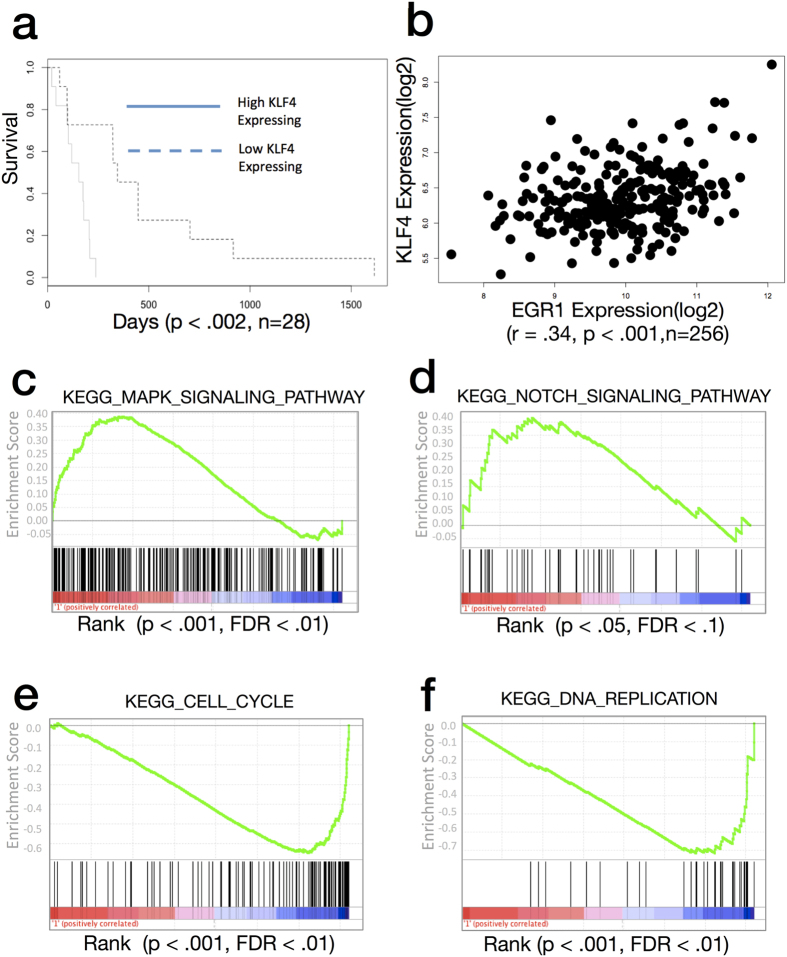
Increased KLF4 expression is associated with enhanced GBM tumor aggressiveness *in-vivo*. (**a**) Dose-dependent Association of KLF4 mRNA expression level with patient survival in the Proneural GCIMP- clinical subtype. The Kaplan Meier estimate of survival was performed on TCGA Clinical GBM samples in the Proneural GCIMP- clinical subtype (n = 28), which is the subtype of the parental tumors for all GSC lines used in the current publication. High KLF4 expressing samples were defined by >= mean(KLF4) + 0.0.5*SD(KLF4) and low KLF4 expressing samples were defined by <= mean(KLF4) − 0.5*SD(KLF4). (**b**) KLF4 and EGR1 mRNA expression levels correlate across clinical GBM Samples. The Pearson correlation coefficient was computed on KLF4 and EGR1 mRNA levels (Affymetrix Exon Array Platform) on 256 TCGA clinical GBM samples (r = 0.34, p < 0.001). (**c–f**) Geneset Enrichment Analysis (GSEA) of high KLF4 expression vs. low KLF4 expressing Proneural GCIMP- tumors for MAPK, NOTCH, KEGG_CELL_CYCLE, and KEGG_DNA_REPLICATION.

Interrogation of gene-expression microarray data for 49 clinical GBM samples with the same molecular subtype as the 5 GSCs used in the current study (Proneural GCIMP-) revealed that KLF4 expression significantly predicted survival (Fig. 8a). KLF4 expression levels correlated negatively with survival using the Pearson Correlation Coefficient. (n = 49, r = −34, p < = 0.01). A Kaplan Meier model was fit between high-expressing KLF4 tumors and showed a significant effect on survival (p < 0.002, n = 28).

In agreement with our observations *in-vitro*, EGR1 expression levels in these clinical samples were significantly correlated with KLF4 expression levels (n = 49, r = 0.32, p < 0.01) as well as for all clinical subtypes (n = 256, r = 0.34, p < 0.001).

Next, we examined pathway activation in high vs. low KLF4 expressing samples (n = 28) using 186 KEGG genesets from the GSEA collection. In support of our *in-vitro* findings, samples with the highest levels of KLF4 showed upregulation of MAPK (p < 0.001, FDR < 0.01) and NOTCH (p < 0.05, FDR < 0.1) genesets compared to the lowest KLF4 expressors. Further, the highest KLF4 expressors showed downregulation of CELL_CYCLE (p < 0.001, FDR < 0.01) and DNA_REPLICATION (p < 0.001, FDR < 0.01) genesets, consistent with a partial reduction in cellular proliferation and increase in anchorage-independent growth seen with *in-vitro* KLF4 overexpression (Fig. 7c,d). These results suggest a similar effect of increased KLF4 levels to what has previously been observed in multiple myeloma, in which higher KLF4 levels reduce proliferation *in-vitro*, but produce tumors that are associated with decreased patient survival *in-vivo* due to increased resistance to apoptosis by alkylating chemotherapy agents^21^. The role of KLF4 in this process is further supported by our ChIP-Seq data that indicate binding near the promoters of genes in GSC involved in the negative regulation of apoptosis (Fig. 6c).

**Figure 9:**
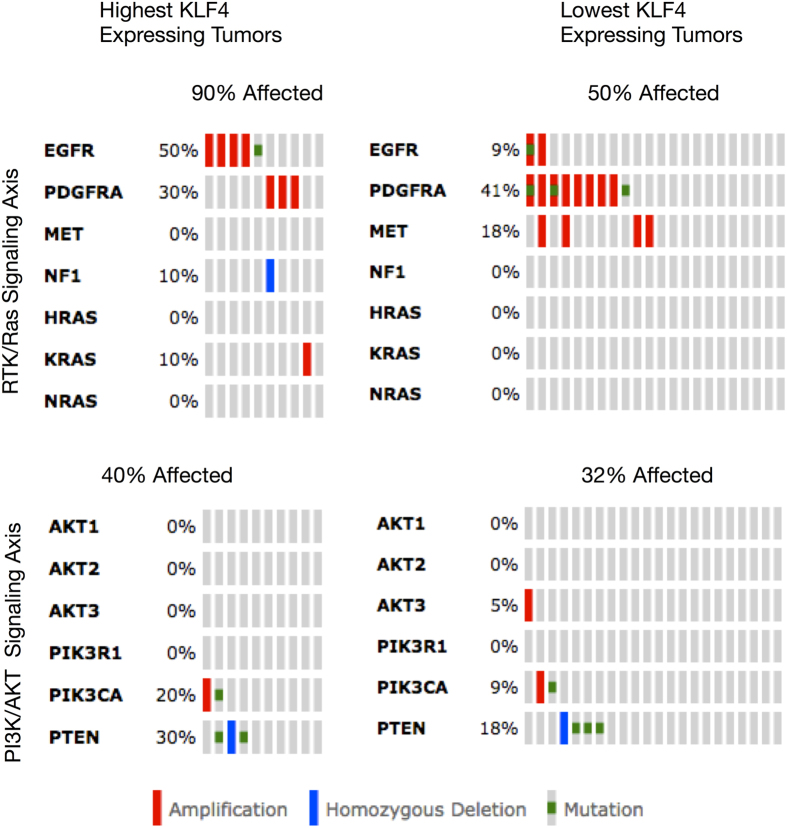
Genomic Alterations from Proneural GCIMP- Human GBM tumors associated with High and Low KLF4 Expression. High KLF4 expressing Proneural GCIMP- tumors show significantly more genomic alterations to the RAS/MAPK signaling axis (fishers exact test, p < 0.01) than low KLF4 expressing Proneural GCIMP- tumors.

A difference between MAPK activation for the two groups of clinical samples suggests that genomic alterations to genes in the RTK/Ras signaling axis upstream of MAPK might vary between the two groups. Indeed, we observed that 90% of high KLF4 expressing tumors in the GCIMP- Proneural subtype shown alteration in RTK/Ras genes (EGFR, PDGFRA, MET, NF1, RAS) compared to only 50% in the low KLF4 expressing tumors (p < 0.05, two-tailed Fishers Exact Test). Differences between the 2 groups of clinical samples for genomic alterations in the PI3K/AKT signaling axis (AKT1, AKT2, AKT3, PIK3R1, PIK3CA, PTEN) were not statistically significant (Fig. 9).

## Conclusion

By conducting a computational analysis of global gene expression changes in GSC and NSC, we have shown that both cell types show a substantial overlap in regulatory circuitry during differentiation. Our data identify both previously characterized regulators in NSC/GSC biology such as SOX2, OLIG2, DLL, NOTCH, and HES1, and reveal a new role for the transcription factor KLF4 in GSC differentiation. As reported by Qin *et al*.^22^, overexpression of KLF4 in neural stem cells both blocks *in-vitro* differentiation and reduces proliferation^22^. We have observed an identical effect of KLF4 overexpression in GSC. Reduction of proliferation and increase of anchorage-independent growth is consistent with the promotion of stem-like phenotype, and we suggest that the role of KLF4 in glioblastoma mirrors that in Multiple Myeloma, where increased KLF4 levels are associated with an overall reduction of proliferation, but greater resistance to alkylating chemotherapy agents^21^.

Our data support a model in which KLF4 is activated in GSC downstream of the MAPK pathway in part through EGR1, and we further demonstrate that EGR1 acts as a transcriptional regulator of KLF4 in GSCs *in-vitro*, and its expression level positively correlates with KLF4 in clinical GBM tumor samples. KLF4 acts as a central node in our model to regulate several key transcription factors and pathways, including SOX2 and DLL/NOTCH. A positive feedback loop is also formed from KLF4 back to the MAPK pathway through the upregulation of HRAS. The concomitant downregulation of HES1 and upregulation of PTEN during NSC differentiation raises the possibility that KLF4 may also act to indirectly suppress PTEN activity, a known deletion target in approximately forty percent of human GBMs. The positive regulation of the PI3K/AKT signaling axis through suppression of PTEN by HES transcription factors has also been documented in other cancers such as T-ALL^23^. This relationship may be the focus of future investigation.

Given that both KLF4 and EGR1 play roles in the differentiation of normal neural progenitors^21^,^24^, what might allow them to promote a persistent undifferentiated state in glioblastoma stem cells? Both transcription factors have been shown to function as tumor suppressors or oncogenes depending on cellular context^25^. The activity of KLF4 is normally constrained in part by the activation of p21, which induces cell-cycle arrest^26^. The repression of p21 by MYC, previously demonstrated in GSCs, may allow KLF4 to escape this restriction. In a similar way, EGR1 transcriptionally activates both PTEN and P53, and these two tumor suppressors act to limit its oncogenic capacity. The genomic loss of PTEN/P53 activity, frequently seen in glioblastoma, removes these critical negative feedback points (including p21 activity), allowing EGR1 to act potentially without restraint on downstream target genes^26^. *Calogero et al.*^27^ reported that EGR1 levels are suppressed in glioblastoma (n = 31) compared to normal brain tissue while Mittelbronn *et al*.^28^ found that EGR1 was upregulated in all grades of astrocytomas compared to normal glial cells *in situ*, we show using a larger number of samples (n = 256) that EGR1 mRNA levels vary greatly between different tumors and are highly expressed in many samples.

Future work may define how KLF4 is involved in other aspects of GBM biology such as its cerebral invasive nature, as suggested by a previous study that showed EGR1 transcriptionally regulates KLF4 to induce *in-vitro* cell-scattering, in part, by suppressing the expression of E-Cadherin in 2 different established cell-lines^29^.

## Methods

### Tissue Culture

Fifteen cm Tissue Culture Dishes were coated with poly-ornithine (Sigma P4957) for 1 hr at 37 °C and washed 3 times with PBS. Cells were plated at 1E6 cells per dish in NBE media (Tumor stem cells derived from glioblastomas cultured in bFGF and EGF more closely mirror the phenotype and genotype of primary tumors than do serum-cultured cell lines). After 3 to 5 days in NBE media, when cells reached 70% confluence, cells were processed for RNA collection as Control sample at Day 0 or media was changed to DMEM (Invitrogen)/5%FBS (Invitrogen)/2 μM all trans-retinoic acid, RA (Sigma R2625). Cells treated with RA were processed on Day 3 and Day 10. RNA was collected using Qiagen RNeasy Kit (74106). Cells were lysed directly on the dishes and total RNA was isolated following the manufacturers protocol. Media was changed approximately every 2–3 days to maintain a constant dose of RA. All experiments were done in triplicate.

### Total RNA extraction

RNA was prepared using TRIzol^®^ reagent and PureLink^®^ RNA Mini Kit (Life Technologies, Carlsbad, CA) RNA quantity was determined using the NanoDrop^®^ ND-8000 spectrophotometer and quality was assessed using the Agilent Bioanalyzer 2100 system.

### Affymetrix GeneChip^®^ Human Genome U133 Plus 2.0 Array

500 ng of total RNA was prepared for hybridization using the Affymetrix 3′ IVT Express Kit Arrays were processed following manufacturer’s recommendations using the Affymetrix GeneChip^®^ Hybridization Oven 640, Fluidics Station 450 and Scanner 3000 7G.

### DNA extraction

High molecular weight genomic DNA was prepared using a QIAamp DNA Kit (Qiagen, Valencia, CA) following the manufacturer’s instructions.

DNA quantity was determined using the NanoDrop^®^ ND-8000 spectrophotometer and quality was assessed by electrophoresis on a 2% agarose gel.

### Analysis of Gene-Expression Microarray Data

Affymetrix 133 Plus 2.0 CEL files were processed using the MAS5 algorithm and probesets were converted to Refseq Transcript ID using a custom Chip Description File (CDF). Differentially expressed genes were computed for both 0–3 days and 0–10 days for each cell-line using an independent 2-tailed Students T-test. P-values were corrected using the method of Benjamini and Hochberg for conversion to a False Discovery Rate. For 5 cell-lines that showed evidence of differentiation based on protein markers, a two-way analysis of variance was used to determine common genes that were changing significantly across days 0, 3, and 10. ANOVA p-values were corrected using the method of Benjamini and Hochberg.

### Transcription Factor DNA-Binding Motif Enrichment Analysis

Transcription factor dna-binding motifs were taken from the Swissregulon database (version 3). Swissregulon motifs were originally curated from both the Transfac and JASAPAR public motif databases as well as from selected publicly available ChIP-Chip and ChIP-Seq data sources. The Swissregulon database has the additional advantage that motifs are filtered by evolutionary conservation among 7 placental mammals using the MONTEVO algorithm. Evolutionary filtering increases the likelihood that any given motif shows functional binding to its cognate transcription factor. In addition, the use of evolutionary conserved motifs makes the comparison of regulatory networks between species (Human and Mouse) less problematic, as regulatory binding motifs can experience evolutionary drift. Motifs associated with each Refseq transcript (Hg18) were selected that occurred between −500/+100 relative to each transcription start-site of each gene as annotated in the Refgene.txt file from the UCSC genome browser database. Although important regulatory binding events do occur outside the proximal promoter region of each gene, this region was chosen as a reasonable compromise between sensitivity and selectivity to represent the relationship between transcription factor binding and gene-expression, an approach that was previously used in the successful reconstruction of regulatory networks in a myeloid leukemia cell-line^30^.

Motif enrichment for the set of differentially expressed genes was computed by first creating a motif score for the promoter of each Refseq transcript. This score was created as the sum of the posterior probabilities for each motif for each gene and reflects both the number of motifs in each promoter as well as their evolutionary conservation. A composite score for each motif type in the set of differentially expressed genes was then computed, where ***n***is the total number of differentially expressed genes, ***m*** is the number of motifs in the promoter of each genes (−500, +100 TSS) and ***s*** is the evolutionarily conserved motif score.

Significance of the composite score for each motif was computed by permutation. 10,000 same-sized samples of genes were chosen randomly from the dataset and motif enrichment scores were computed for each randomly chosen set of genes. P-values were computed from this null-distribution using the Empirical Cumulative Null Distribution (ECDF) and converted to false discovery rate using the method of Benjamini and Hochberg.

### Geneset Enrichment Analysis

For both the GSCs and Murine NSCs E14, the F-test statistic from a two-way ANOVA for the effect of treatment day (0, 3, 10) was used to rank genes from the microarray. Genesets representing canonical pathways (1452) were taken from the C2 database from the GSEA website (Broad Institute). The *GeneSetTest* function of the Limma R package was then used to compute a p-value for the mean rank of each geneset, which uses the Wilcoxon two-sample rank test according to the mean-rank gene-set enrichment method developed by Michaud *et al*.^31^. P-values were corrected for multiple hypothesis testing by the method of Benjamini and Hochberg.

### Gene Regulatory Network Reconstruction

The relationship between transcription-factor binding motifs and gene-expression changes was used to reconstruct a gene-regulatory network. To accomplish this goal, a multivariate regression model was fit between TF DNA-binding motifs and differentially expressed genes using the Random Forest algorithm (*randomforest* R package). Random Forest implements an ensemble of regression trees, and has the advantage over multiple linear regression in that it can model the multiplicative variable interactions and non-linear relationships that can occur between transcription factors^32^.

Links between TF DNA-Binding motifs and targets genes were established using the case-wise *Variable Importance* metric from Random Forest. This metric measures the drop in the performance (measured by mean-squared-error) of the regression model when individual input variables (TF DNA-Binding motifs) are permuted. A significance threshold for motif and target gene relationships was established by randomly permuting the input variables and output variables and fitting a model to establish a null distribution of *Variable importance* values that would be expected by chance. P-values were generated using the empirical cumulative null distribution (ECDF) and corrected for multiple hypothesis testing using the method of Benjamini and Hochberg.

Mean of Squared Residuals for Random Forest:


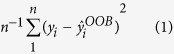


Where *n* is the number of trees in the model and 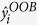 is the mean of ***Out of Bag*** predictions for the *i*th tree. The percent variance explained by the Random Forest model is then:


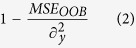


### Cell Culture for shRNA Stable Cell Lines

GSCs were cultured in NBE media consisting of neurobasal media (Invitrogen), N2 and B27 supplements (0.5× each; Invitrogen), and human recombinant bFGF with EGF (25 ng/ml each; R&D Systems). For differentiation studies, cells were cultured in NBE media with 1% fetal bovine serum (Invitrogen) and 2 μM all trans-retinoic acid, RA (Sigma R2625) in poly-Ornithine coated plates during 3 and 10 days.

### Reagents

The following antibodies were purchased: GFAP (Z0334) from DAKO; Nestin (N1602) from IBL; TuJ1 (MMS-435P) from Covance; Sox2 (MAB2018) from R&D Systems; KLF4 (4038), ERK (4695), and p-ERK (9106) from Cell signaling; Actin (A3853) from Sigma-Aldrich; p53 (sc-126), c-Myc (sc-40) from Santa Cruz. The following plasmids were purchased: pLenti4/TO/V5-DEST and ViraPower mix from Invitrogen; pLVX-Tet-On 3G (631354); pLM-mCherry-KLF4 (23243) pCMV-VSV-G (8454) and pCMV-dR8.2 dvpr (8455) from Addgene; KLF4 ShRNA (RHS4533-NM_004235) and EGR1 ShRNA (RHS4533-NM_001964) from Openbiosystem.

### Confocal Analysis

Cells were fixed with 4% PFA for 30 min at 4 °C, permeabilized with 0.5% Triton X-100 in PBS and blocked with 1% BSA, and then stained with primary antibodies followed by Alexa Fluor-conjugated secondary antibodies (Invitrogen). The cells were then examined under either a light or fluorescence microscope (Zeiss).

### Cloning and Virus Preparation

KLF4 gene was inserted into Gateway vector pCR8⁄GW⁄TOPO and subsequently into pLenti4/TO/V5-DEST. Additionally, the KLF4 gene was inserted into a pLVX-Tet-On 3G doxycycline-inducible vector (Clontech), downstream of both a turbo GFP gene and an IRES sequence, respectively. Lentivirus was produced in 293FT cells with packaging mix (ViraPower Lentiviral Expression Systems for KLF4 lentivirus from Invitrogen; pCMV-VSV-G and pCMV-dR8.2 dvpr for ShKLF4 and ShEGR1 from Addgene). Plasmids were transfected with Lipofectamine and Plus reagent (Invitrogen). After 3 hrs, media was changed with 5% FBS. Virus-laden supernatant was collected at 24, 48 and 72 hrs. The supernatant was filtered and concentrated by ultracentrifugation, and viral titer was determined by serial dilution.

### Limiting Dilution and Proliferation Assay

*In vitro* NBE-cultured GSCs were dissociated into single-cell suspensions. For the limiting dilution assay, cells were plated into 96-well plates at various seeding densities (2, 5, and 10 cells per well) and were incubated at 37 °C for 1–2 weeks. Each well was examined for the formation of tumor spheres. For the proliferation assay, cells were plated into 6-well plates with 2 × 10^5^ cells per well and incubated at 37 °C for 2 and 4 days. At the time of quantification, cells in each well were counted using Vi-CELL XR (BCM).

### Western Blot

Subcellular fractions were prepared with the ProteoExtract subcellular proteome extraction kit (Calbiochem) following manufacturer’s instructions. Nuclear and total soluble cell-fraction lysates were quantified using the Pierce BCA protein assay (Thermo Scientific). Proteins were separated by SDS-PAGE on a NuPAGE mini gel (Invitrogen). Proteins were transferred to a PVDF membrane, blocked with 5% nonfat dry milk or Bovine Serum Albumin (Sigma) in TBST for 1 hr RT, primary antibody was added and incubated O/N at 4 °C. Then blot was washed 3X in TBST, then incubated in 5% nonfat dry milk in TBST with secondary-HRP conjugated antibody for 1 hr RT, washed 3X in TBST, developed with SuperSignal West Pico chemiluminescence kit (Thermo Scientific) and exposed to Biomax MR film (Kodak). Western blots were performed with the following antibodies: anti-beta actin (Sigma), anti-EGR1 and anti-Hes1 (Santa Cruz), anti-GFAP and anti-Histone H3, anti-KLF4, anti-SOX2 (R&D), anti-nestin (Covance), anti-beta-tubulin (Sigma).

### ChIP-Seq of KLF4

Following the Active Motif ChIP-It Express kit with modifications, cells were seeded on 15 cm polyornithine-coated plates. When they reached 70–80% confluency (approximately twelve million cells/plate), cells were fixed with a formaldehyde solution for 8 min at 37 °C, reactions were stopped with glycine solution for 5 min RT, cells were harvested by scraping plates on ice with cold PBS and collecting cell pellets by centrifugation. Cells were sonicated in Active Motif shearing buffer with fresh protease inhibitors and PMSF using the following Covaris SonoLab 7.1 protocol: peak incident power 240, duty factor 20, cycles/burst 200, duration 300 sec. To assess the efficiency of DNA shearing and determine initial DNA concentration, an aliquot of sheared chromatin was subjected to crosslink reversal and treated with proteinase K. DNA was purified by phenol/chloroform extraction and EtOH precipitation. An aliquot of the DNA was separated by electrophoresis through a 1.5% agarose gel to determine the shearing efficiency. For the chromatin immunoprecipitation reaction: 100 μg sheared chromatin + 10 μg biotinylated anti-human KLF4 antibody (or biotinylated normal IgG) (R&D) + 40 μl streptavidin magnetic beads (R&D) were mixed in Active Motif ChIP buffer 1. Samples were incubated with end-over-end shaking for 24 hr at 4 °C, beads were washed 3X, chromatin was eluted, subjected to crosslink reversal and treated with proteinase K. DNA was purified by phenol/chloroform extraction and EtOH precipitation. Finally, DNA pellets were resuspended in 45 μl of TE, quantified using High Sensitivity DNA Bioanalyzer chips (Agilent Technologies), and analyzed by DNA sequencing.

### ChIP-PCR

After chromatin immunoprecipitation reaction, 5 μl of each DNA sample were added per well in 96 well plate for RT-qPCR. Each sample was run in triplicate and a standard curve was prepared for each primer pair using input DNA. Input DNA refers to the purified DNA after sonication, but before the ChIP reaction. EpiTect ChIP qPCR primers for specific promoters were purchased from Qiagen. Fast SYBR green master mix was from ABI. Fold enrichment was calculated by finding the slope of the standard curve, solving for the DNA quantity of each sample, and finally determining the fold enrichment of the ChIP sample relative to the IgG sample.

## Additional Information

**How to cite this article:** Riddick, G. *et al*. A Core Regulatory Circuit in Glioblastoma Stem Cells Links MAPK Activation to a Transcriptional Program of Neural Stem Cell Identity. *Sci. Rep.* **7**, 43605; doi: 10.1038/srep43605 (2017).

**Publisher's note:** Springer Nature remains neutral with regard to jurisdictional claims in published maps and institutional affiliations.

## Supplementary Material

Supplementary Information
