# Supplementary material for: A Core Regulatory Circuit in Glioblastoma Stem Cells Links MAPK Activation to a Transcriptional Program of Neural Stem Cell Identity
Source: Sci Rep. 2017 Mar 3;7:43605. doi: 10.1038/srep43605 (PMC5335262; doi:10.1038/srep43605)

# A Core Regulatory Circuit in Glioblastoma Stem Cells Links MAPK Activation to a Transcriptional Program of Neural Stem Cell Identity

Supplementary Data

Gregory Riddick*,Svetlana Kotliarova*,Virginia Rodriguez*, Kim HS*,

Amanda Linkhous, Andrew Storaska, Susie Ahn, Jennifer Walling, Galina Belova, and Howard A. Fine

*Co-first authors

Supplementary Figure Legends.

S1. Transcription factor motif enrichment analysis of global gene expression changes from the differentiation of 5 GSC lines.

S2. Western blot of KLF4,EGR1,NESTIN, and GFAP in established glioma cell line U87 in control vs overexpression conditions(EGR1,KLF4) under differentiating conditions (RA/FBS).

S3. Enriched Transcription Factor Motifs During NSC E14 Differentiation.

S4. Enriched Transcription Factor Motifs During Differentiation of 5 GSC.

S5. Pathway Analysis P-values for 3 Human NSC Cell-Lines.

S6. Full Gel Images for Figure 1.

S7. Full Gel Image for Figure 2.

S8. Full Gel Image for Supplementary figure 2.

S9. Full Gel Image for Supplementary Figure 7 (923).

S10. Full Gel Image for Supplementary Figure 7 (1228).

**
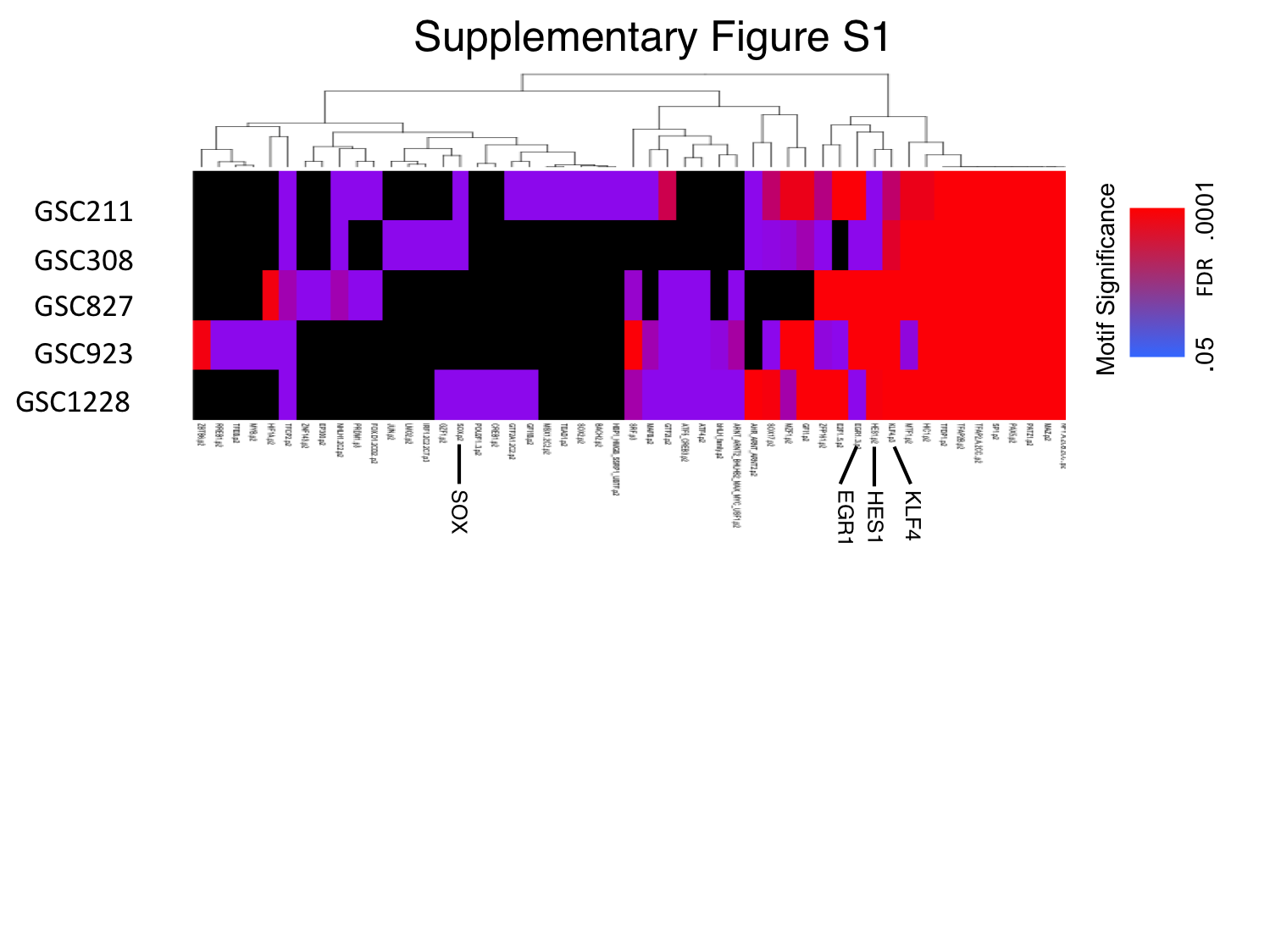
**


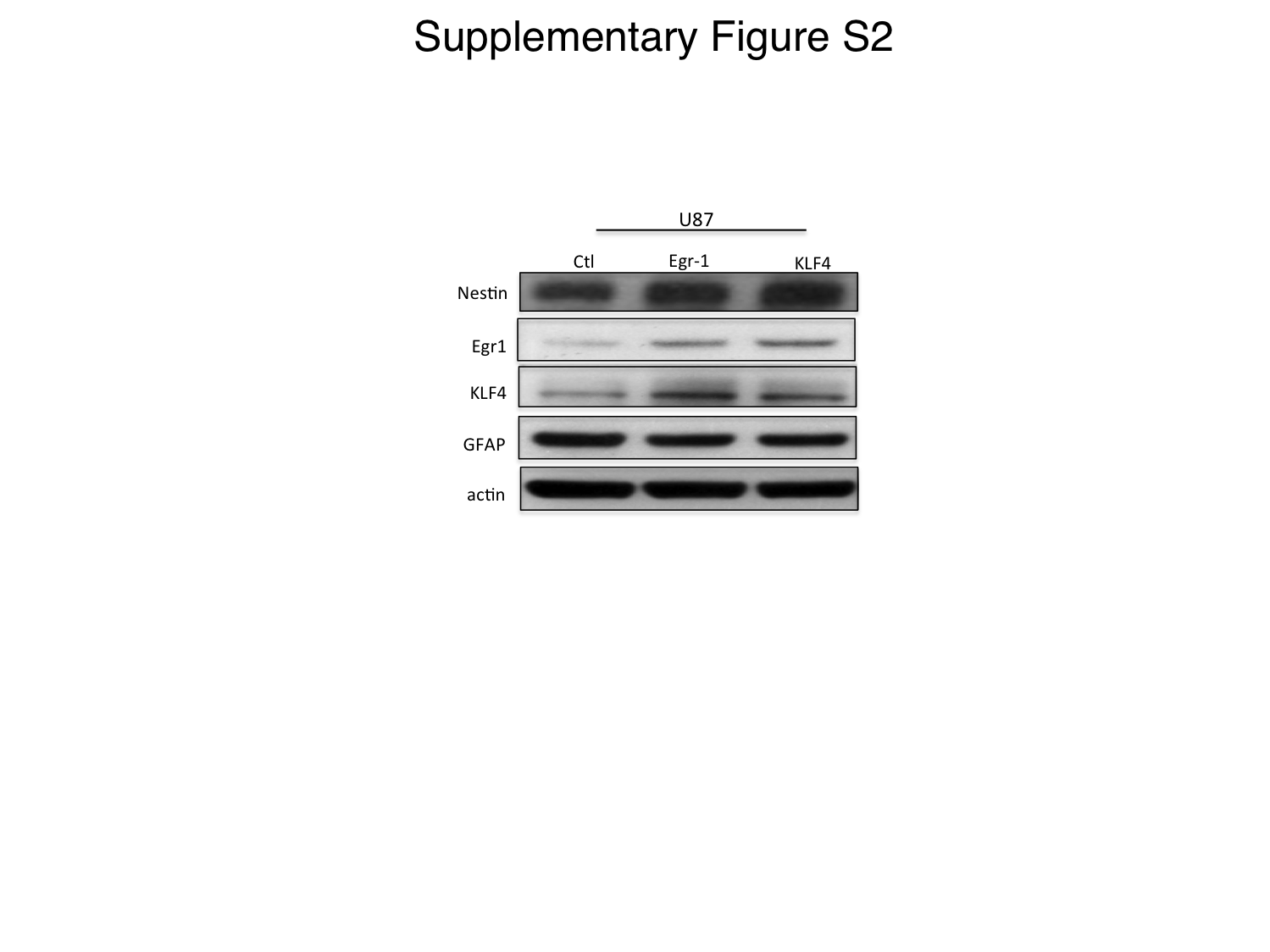


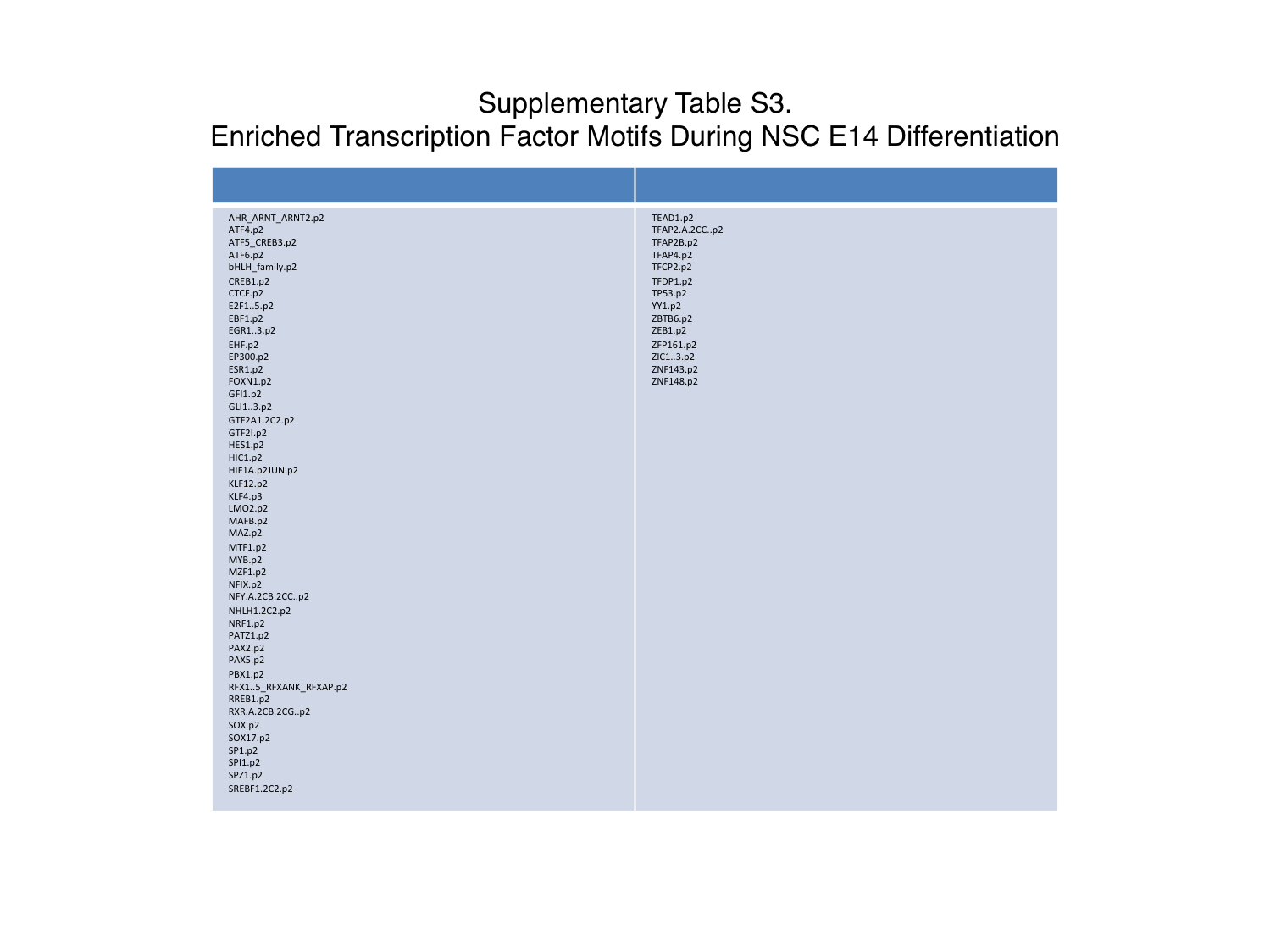


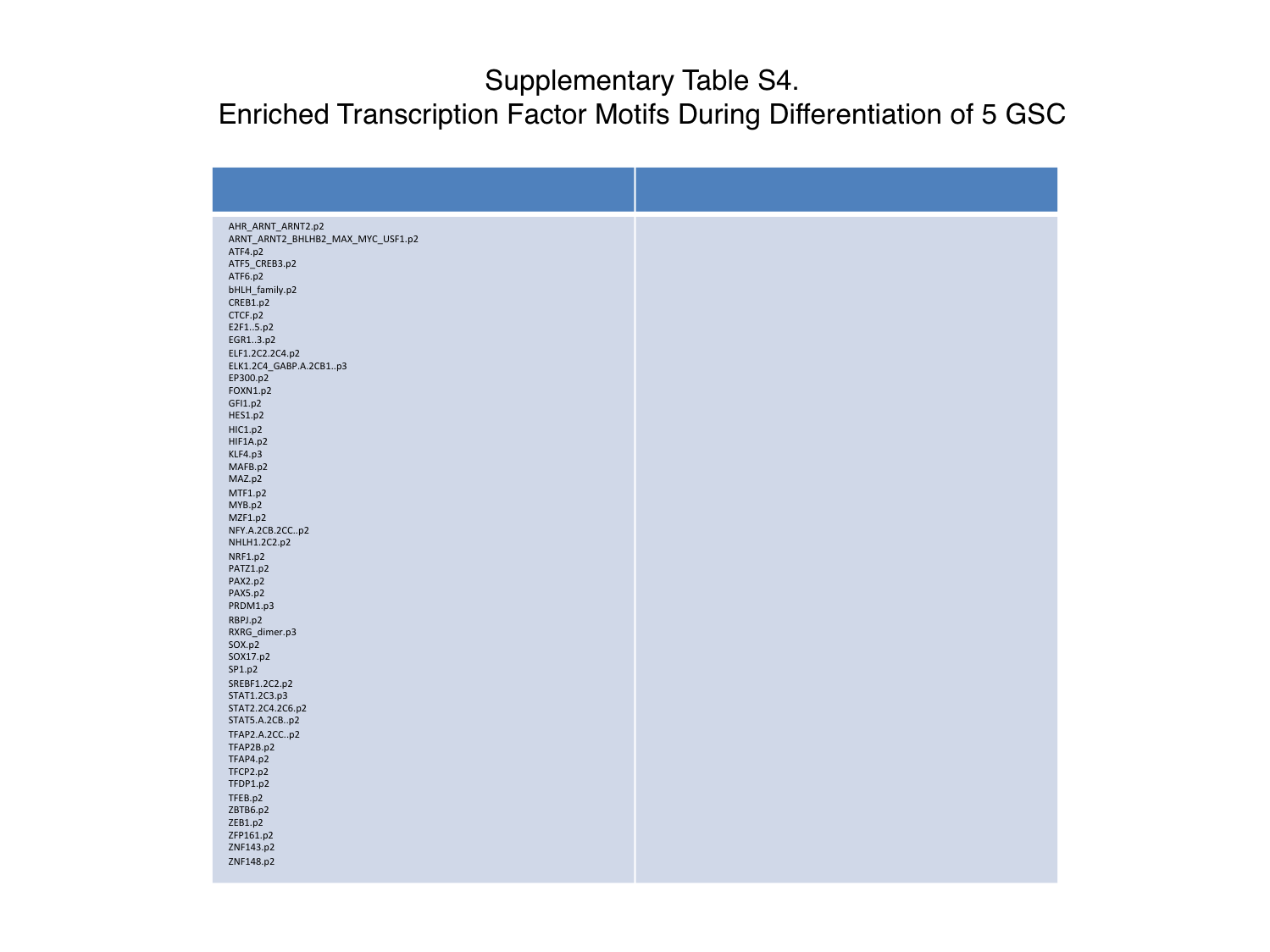


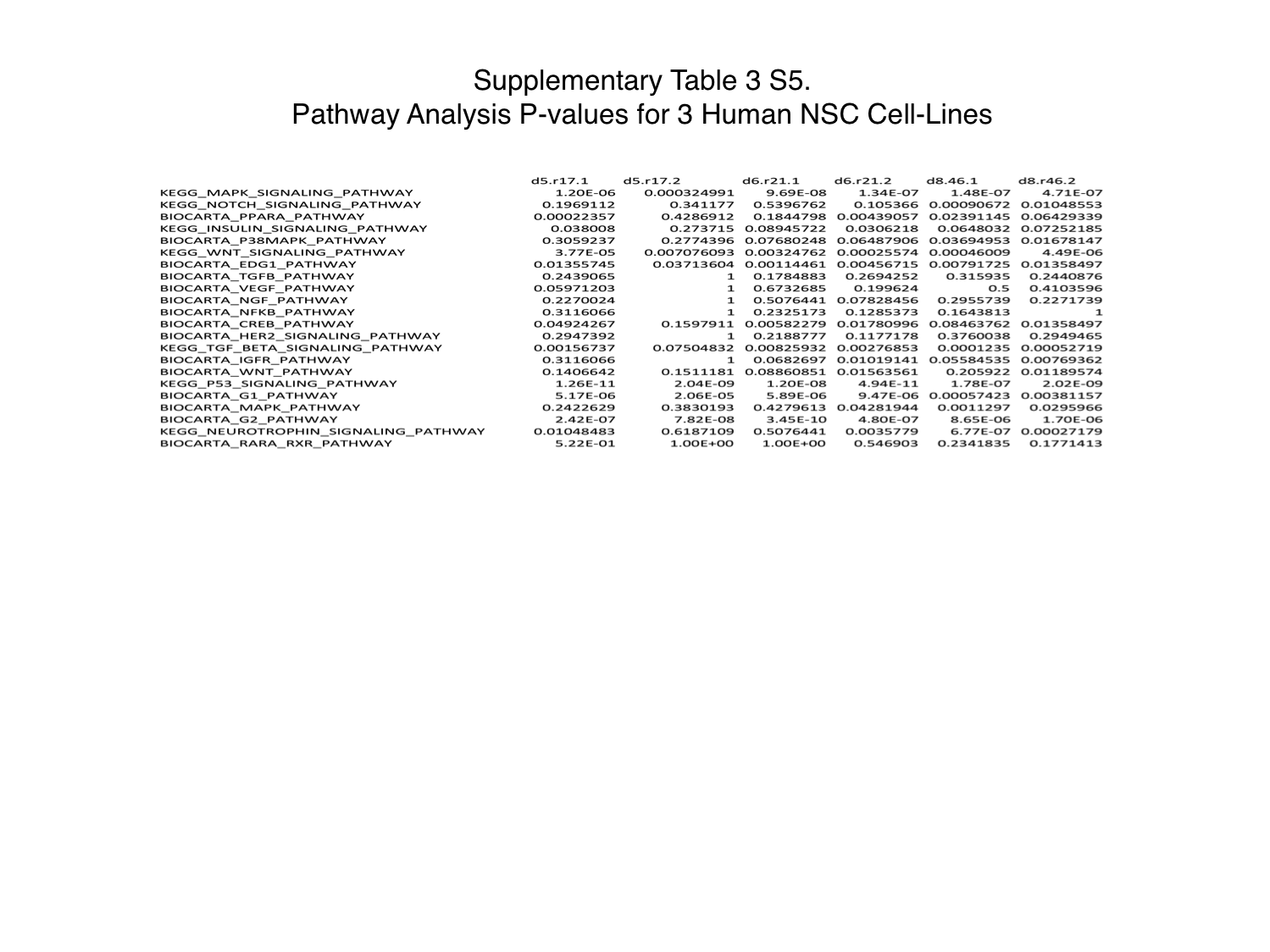


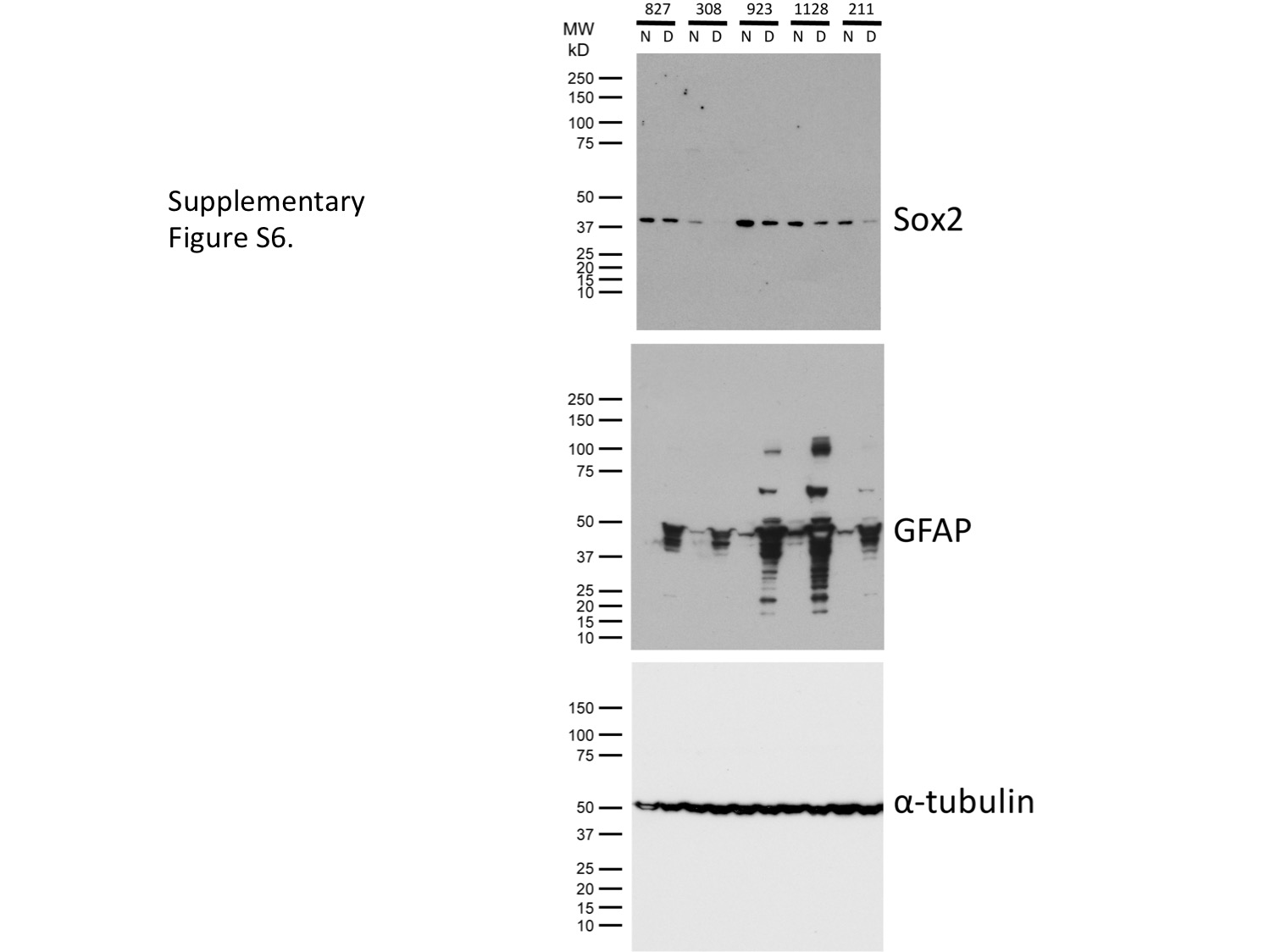


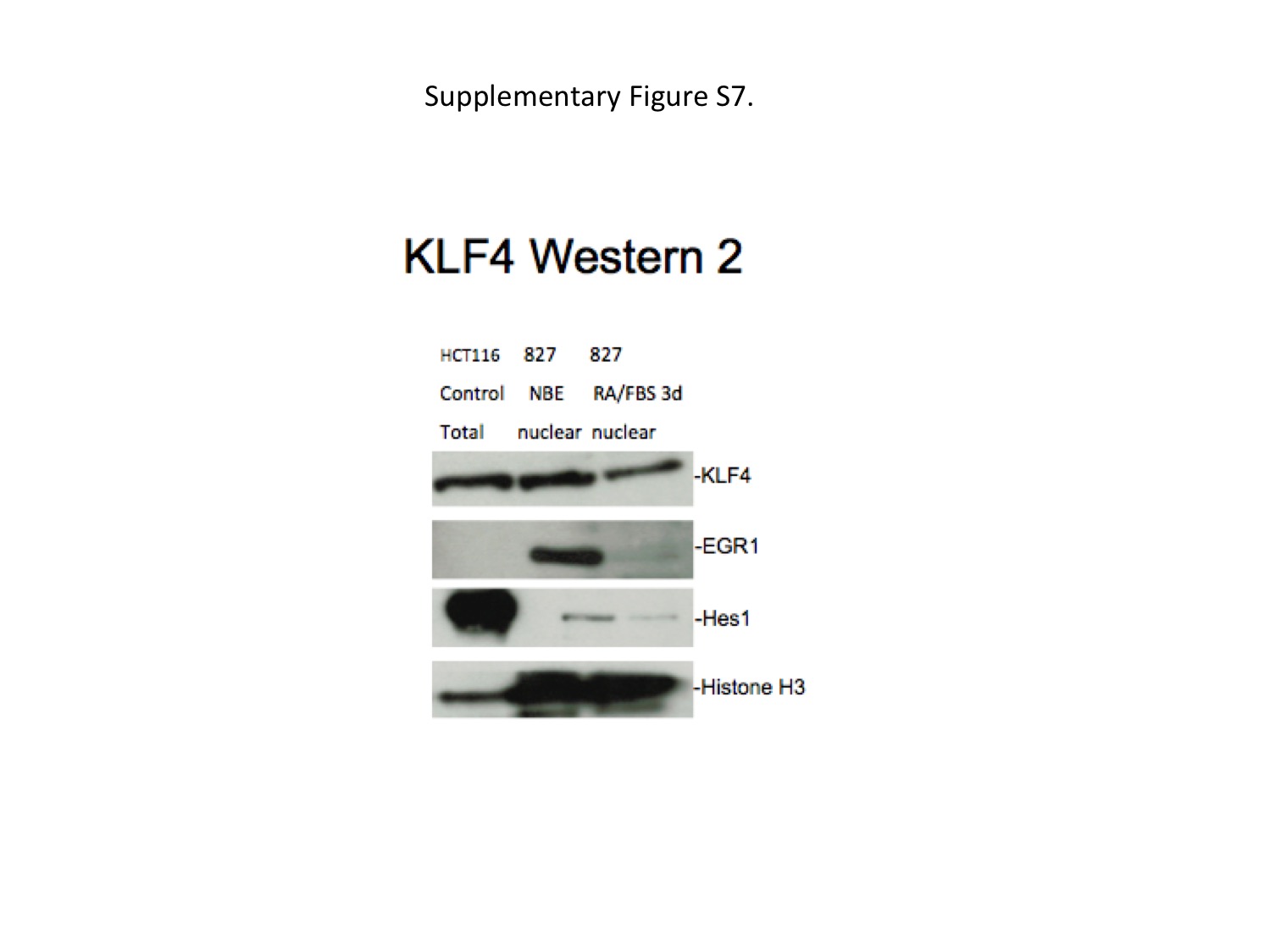


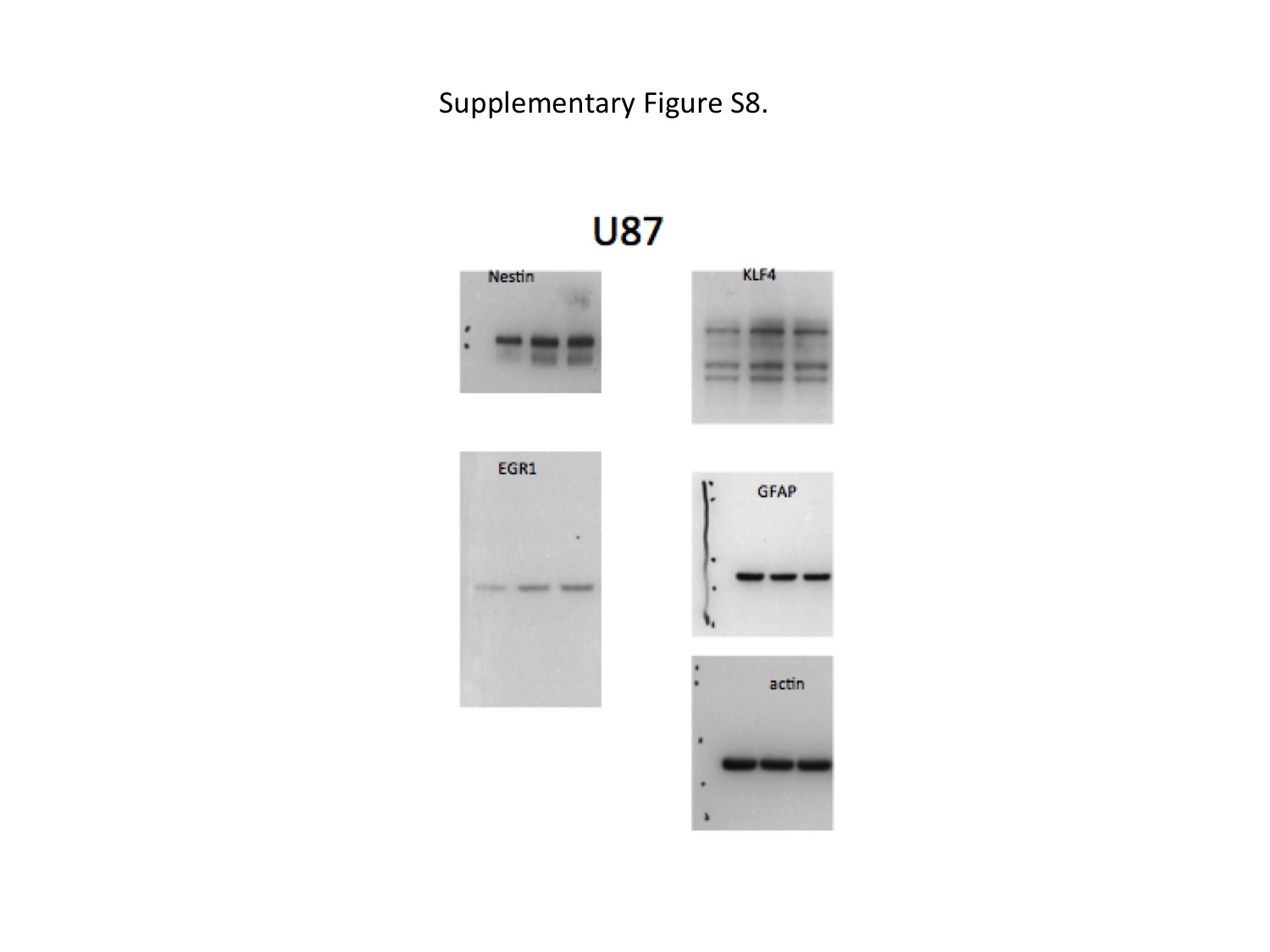


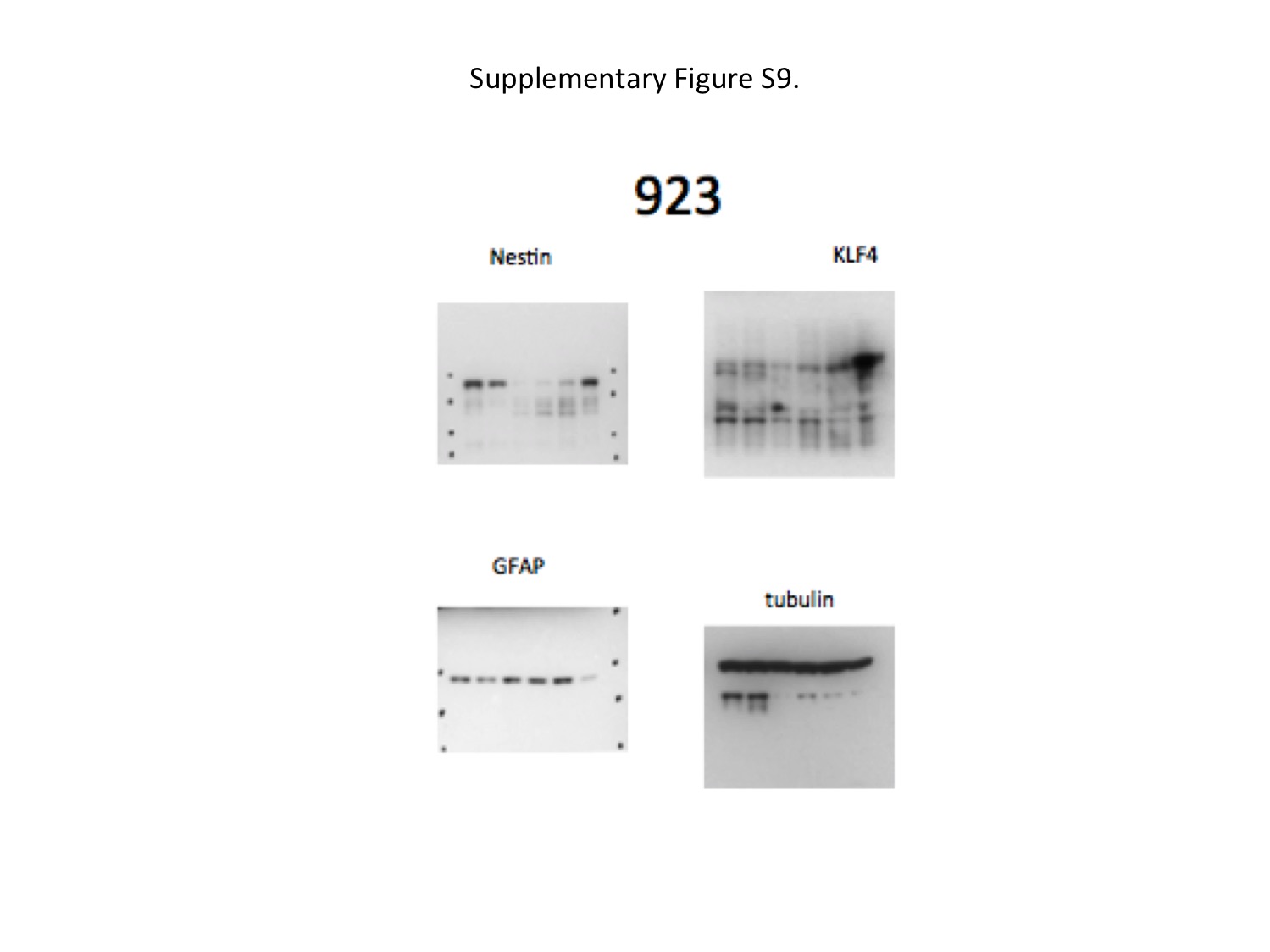


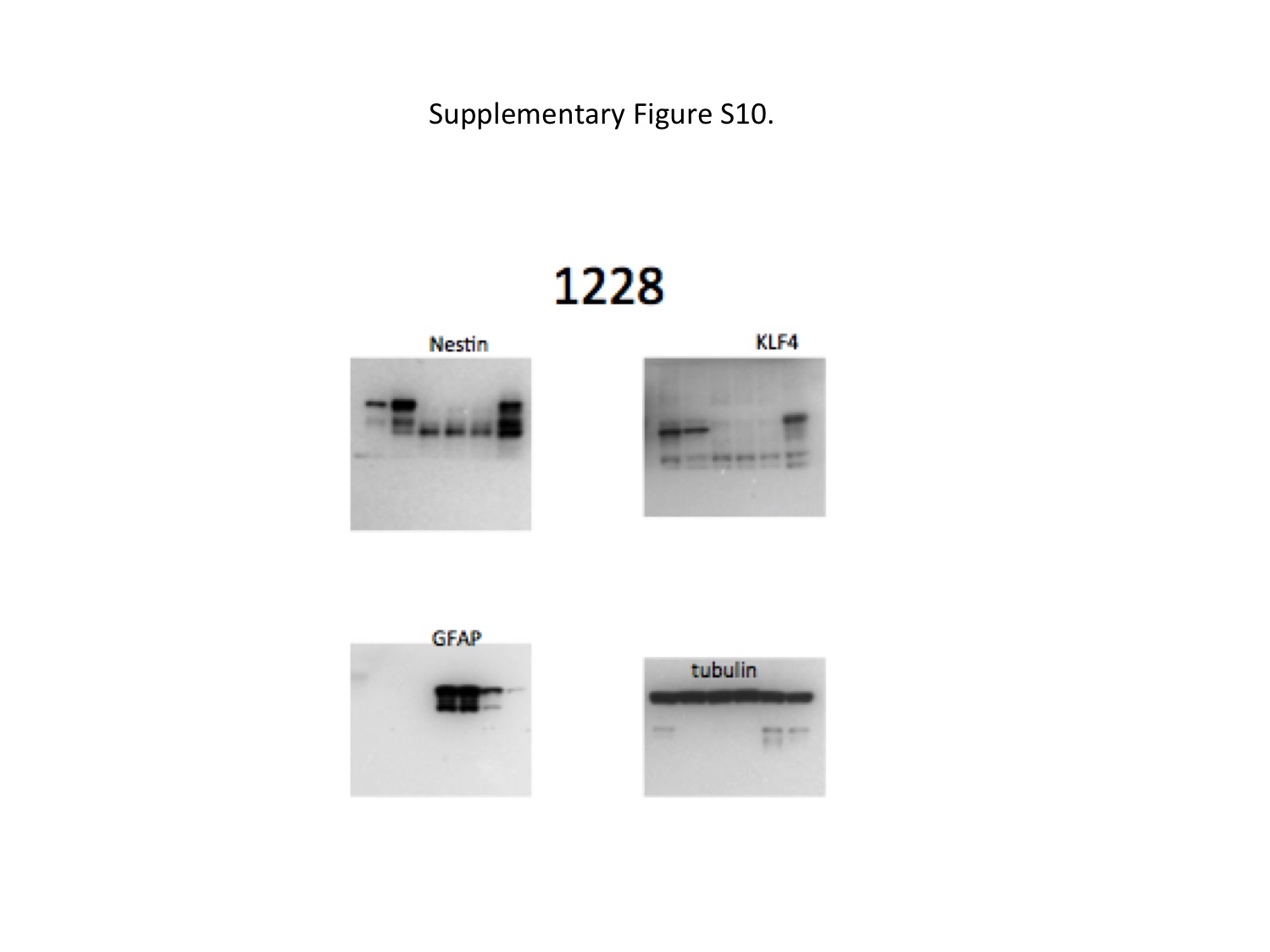

Supplement: Supplementary Information [file srep43605-s1.doc]
